# Phyllotaxy and environmental factors influences on leaf trait dimensions in *Fraxinus mandshurica*: a multidimensional approach within temperate forests

**DOI:** 10.3389/fpls.2025.1626579

**Published:** 2025-07-15

**Authors:** Mingyue Jin, Yunfei Diao, Yunlong Wang, Mingke Zhang, Tianyi Wang, Yajun Ren, Ming Zhong, Wanting Cheng, Chengdong Wang, Honghui Teng

**Affiliations:** ^1^ Jilin Provincial Key Laboratory of Emerging Contaminants Identification and Control, Jilin Normal University, Siping, China; ^2^ Key Laboratory of Environmental Materials and Pollution Control, the Education Department of Jilin Province, Jilin Normal University, Siping, China; ^3^ Jilin Normal University, Siping, Jilin, China; ^4^ Heilongjiang Institute of Ecology, Harbin, Heilongjiang, China; ^5^ School of Ecology, Northeast Forestry University, Harbin, China

**Keywords:** phyllotaxy, environmental factors, economics traits, stomatal traits, *Fraxinus mandshurica*

## Abstract

**Introduction:**

Light and soil nutrients are strong drivers of leaf trait variation, but the relative importance in shaping intraspecific trait variation across leaf developmental gradients remains poorly assessed. Previous studies mostly focused on single traits, while fewer have employed multidimensional trait syndromes framework to explore how plants optimize resource use and maintain physiological homeostasis.

**Methods:**

We measured leaf economic traits (e.g., specific leaf area, leaf nitrogen concentration) and stomatal traits (e.g., stomatal density, size) in leaflets at different phyllotactic positions of the compound-leaved species *Fraxinus mandshurica* in temperate forests of Northeast China, while assessing the effects of phyllotaxy and environmental factors (light, soil nutrients) on these traits.

**Results:**

We found that (1) specific leaf area and leaf nitrogen concentration significantly increase along the phyllotaxy gradient (from phyllotaxy 1 to 6), while leaf dry matter content, leaf thickness, and stomatal density significantly decrease. No significant variation in the dimensions of economic and stomatal traits was observed across the phyllotaxy gradient; (2) phyllotaxy modulates trait coordination, with decoupled economic and stomatal traits at phyllotaxy 1 but coupled relationships at phyllotaxy 2–6; (3) environmental factors had a greater impact on economic trait dimensions variation, whereas phyllotaxy was more important for stomatal trait dimensions.

**Discussion:**

Our study highlights the significance of trait dimensions in understanding plant functional strategies. We emphasize that the influence of environmental factors or phyllotaxy on trait variation is trait-specific, indicating distinct mechanisms for resource acquisition and water use. We recommend considering phyllotaxy when predicting plant responses to environmental changes.

## 1 Introduction

In the context of increasing global climate change and environmental variability, understanding the mechanisms by which plants regulate their traits for resource acquisition and ecological adaptation has emerged as a key focus in the field of functional ecology (Laughlin, 2014; Joswig et al., 2022; Liu et al., 2023). In recent years, functional trait ecology has developed rapidly, providing a theoretical basis for revealing the response mechanisms of plants to environmental gradients and changes in resource distribution (Wang et al., 2022; Carmona, 2023; Garnier et al., 2025). Among them, leaf economic traits, including specific leaf area (SLA) and leaf nitrogen concentration (LN), form the “leaf economics spectrum,” reflecting a continuum of plant strategies from fast–growing to resource–conservative along an ecological gradient (Wright et al., 2004; Da et al., 2025); In addition to these economic traits, stomatal traits-including stomatal density (SD), size, and the stomatal pore index (SPI)–play key roles in regulating plant gas exchange and water loss, thereby influencing the carbon–water balance (Sack et al., 2005; Brodribb and Feild, 2010; Xie et al., 2022). Previous studies have suggested that leaf economic and stomatal traits may be interconnected, reflecting a coordinated strategy for optimizing resource use (Liu et al., 2020; Qian et al., 2025). However, research on these trait relationships in compound–leaved tree species, particularly regarding their responses to environmental gradients and phyllotaxy, is still limited.

“Phyllotaxy” refers to the arrangement of leaves along the main axis of a compound leaf (e.g., pinnate leaf), describing the sequence of their emergence. This arrangement reflects both the developmental stage and the functional maturity of the leaves within an individual plant (Niklas, 1988; Efroni et al., 2010). Leaflets at different phyllotactic positions show significant variation in traits such as leaf size, LN, and thickness (LT). These differences are driven by variation in structural development, physiological condition, and microenvironmental factors, including light availability, air flow, and humidity. These variations, in turn, influence the plant’s photosynthetic capacity and water use efficiency (Koch et al., 2018; Bai et al., 2024; Guo et al., 2025). However, past research has largely concentrated on single traits, often neglecting the functional heterogeneity across trait dimensions along the phyllotactic gradient.

Environmental factors, such as light intensity and soil nutrients, are crucial determinants of trait expression (Liu et al., 2023; 2024). Among these traits, economic traits tend to respond rapidly to environmental variation, reflecting their close link to plant resource acquisition and growth strategies (Fajardo and Siefert, 2018; Joswig et al., 2022; Singh and Verma, 2025). In contrast, stomatal traits, being more structurally constrained, typically exhibit slower and more conservative responses, primarily through adjustments in water transpiration and gas exchange (Wang et al., 2019; Xie et al., 2022). For instance, Sack and Frole (2006) observed in temperate deciduous species that leaf mass per area responds markedly and rapidly to variations in light intensity, whereas changes in SD and stomatal length (SL) are relatively slow, highlighting the differential environmental response mechanisms between these two sets of traits. Similar patterns were also observed by Boisseaux et al. (2024) in Amazonian forests. Furthermore, differences in developmental stages among leaflets may influence their responsiveness to environmental cues, thereby intensifying the divergence of trait–environment relationships along the phyllotactic gradient. Nonetheless, the relative contributions of phyllotaxy–associated developmental regulation and environment–driven plasticity in shaping the variation of economic and stomatal traits remain poorly resolved and warrant further investigation.

The coordination among plant functional traits is considered a fundamental mechanism underpinning resource acquisition strategies and the maintenance of ecological stability (Wright et al., 2004; Qian et al., 2025). Numerous studies at the species and community levels have demonstrated synergistic relationships between economic traits (e.g., SLA, LN) and stomatal traits (e.g., SD, SPI), reflecting an integrated adaptive strategy of plants in balancing carbon gain and water regulation (Carlson et al., 2016; Chen et al., 2021). However, Messier et al. (2017) emphasized that trait integration within individual plants is not static; rather, it may weaken, shift, or decouple across different developmental stages, organ positions, or local environmental conditions, giving rise to a pattern of “asynchronous functional integration.” Therefore, dissecting the stability and regulatory mechanisms of covariation between economic and stomatal traits along the phyllotactic gradient in compound-leaved species with distinct developmental hierarchies is essential for understanding intra–individual functional integration and plant adaptive strategies under environmental heterogeneity. Furthermore, previous studies have often emphasized the independent responses of single traits–such as SLA or SD–while overlooking their functional integration, potentially masking critical patterns of trait coordination underlying plant adaptation. Thus, a multidimensional trait-based approach is critically needed to advance our understanding of plants’ integrated adaptive strategies in heterogeneous microenvironments.

This study focuses on two main questions. First, (1) does phyllotaxy significantly affect single leaf economic and stomatal traits, as well as their trait dimensions? Second, if such an effect exists, do environmental factors or phyllotaxy play a more dominant role in shaping these traits? To investigate these questions, we examined four leaf economic traits, four stomatal traits and their variations in 30 *Fraxinus mandshurica* (Rupr.) trees from temperate forests in Northeast China. Additionally, we measured environmental factors (light intensity, soil nutrients) to evaluate the relative influence of environmental conditions and phyllotaxy on these traits.

## 2 Materials and methods

### 2.1 Study site

The study area is situated in Muling northeastern *Taxus cuspidata* National Nature Reserve (130°07’E, 43°95’N~130°28’E, 44°06’N) in Northeast China. The zonal climatic vegetation of this area is broad-leaved Korean pine forest, located at an altitude of 500-700m, with dark brown soil. The region experiences a northern temperate continental monsoon climate, with cold, dry winters, humid, rainy summers, and significant temperature fluctuations during seasonal transitions. The average annual temperature is 2.8°C, and annual precipitation averages 530 mm, mainly occurring from June to August. The frost-free period lasts about 126 days, and the region receives 2,613 hours of sunshine annually.

### 2.2 Sample design

**Figure 1:**
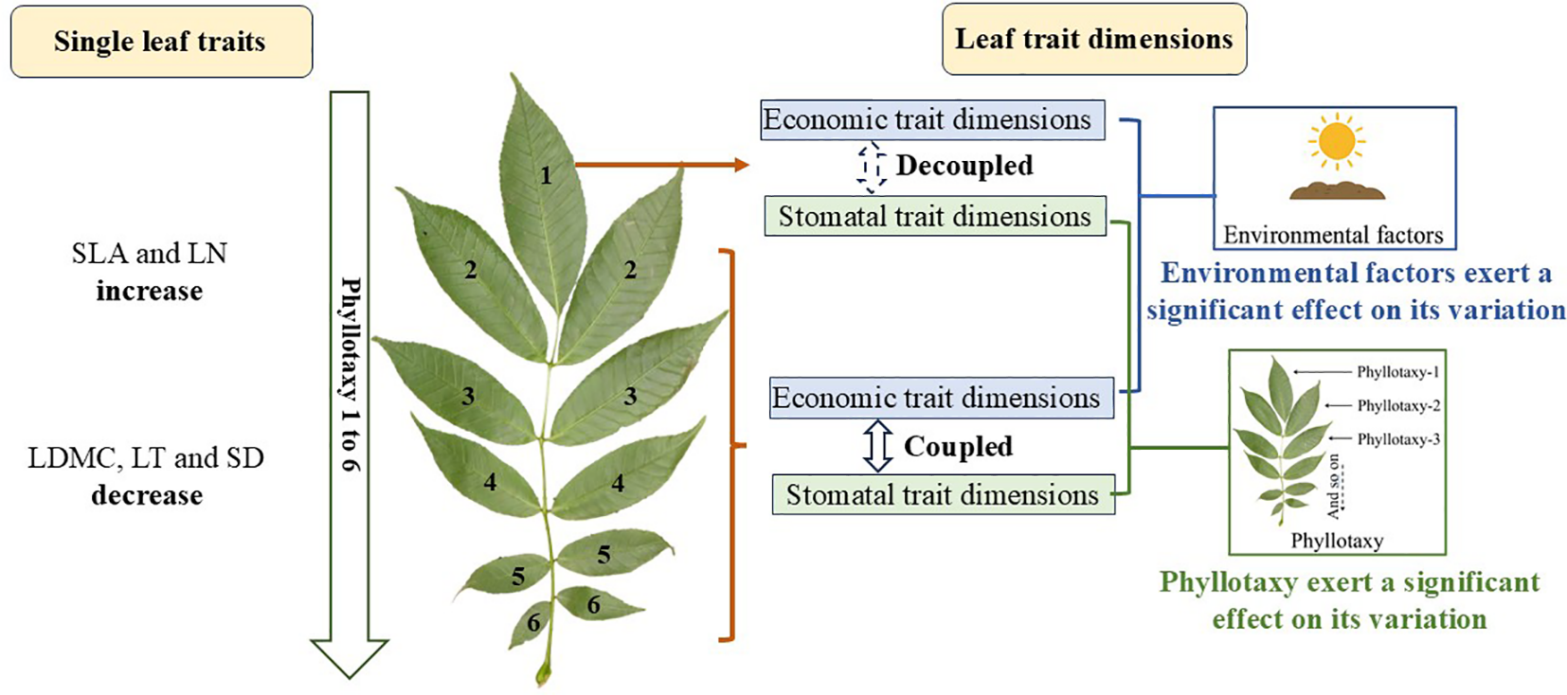
Mechanistic schematic illustrating the effects of phyllotaxy on single-trait variation and the coordination between economic and stomatal trait dimensions, as well as the influence of phyllotaxy and environmental factors on their multidimensional variation. Image from Plant Photo Bank of China (ppbc.iplant.cn).

From mid-July to August 2024, we randomly sampled 30 *Fraxinus mandshurica* (Rupr.) individuals with similar tree height (mean height = 23.52 ± 2.51 m) and diameter at breast height (DBH, mean DBH = 34.13 ± 4.64 cm), all growing under the same slope aspect and comparable slope gradient conditions. To reduce spatial autocorrelation, sample trees were placed at least 10 meters apart. For each individual tree, a trained climber randomly selected a south-facing branch from the upper canopy (Cornelissen et al., 2003). Subsequently, 15–20 mature and fully developed healthy compound leaves were randomly chosen for the measurement of economic traits, while an additional leaf was selected for stomatal trait measurement. The other leaves were used for LN determination. All leaves were collected, sealed in bags, and transported to the lab. Economic traits were measured within 6 hours. The leaves used for stomatal trait measurement were stored in FAA solution (70% ethanol, formalin, and glacial acetic acid in a 90:5:5 ratio). The leaflets of compound leaves need to be classified according to leaflets growth position. As most of the collected leaves after the 6th grade (excluding the 6th grade) are damaged, only the first 6 grade of leaves were collected (**Figure 1**).

For soil samples, litter within a 1–meter radius of each sample tree was removed, and soil was then collected from the top 0–10 cm layer, with three samples taken (with each sample taken at a 120° angle) (Yang et al., 2019). Then, three samples were fully mixed in a bag and sent to the lab for soil nutrient analysis.

### 2.3 Leaf trait measurements

For economics traits: we used scissors to carefully separate the leaflet from the rachis of the compound leaf. We then measured the fresh weight of the leaf with a precision of 0.0001g. Next, we utilized a scanner from BenQ Corporation, China, to capture an image of the leaf area. Subsequently, the image was processed using Photoshop CS 8.01 software from Adobe Systems Inc, USA, allowing us to determine the leaf area with an accuracy of 0.01cm². We measured the LT three times using a micrometer (avoiding the main leaf vein) and took the average value as the LT, with an accuracy of 0.01 mm. The leaves were then dried in an oven until they reached a constant mass (for at least 72 hours at 65°C) and subsequently weighed with a precision of 0.0001 g. Leaf dry matter content (LDMC) was determined by dividing dry weight by fresh weight, while SLA was calculated as the ratio of leaf area to dry weight. For leaves intended for LN measurement, they undergo an initial drying process in an oven, followed by grinding, and then a final drying step to ensure complete dryness. A 1g leaf sample was digested for 40 minutes in a pre-digestion system (H_2_SO_4_+H_2_O_2_). LN was then measured using a Hannon K9840 Automatic Kjeldahl Nitrogen Analyzer (Jinan Hannon Instrument Co., Ltd., Jinan, China).

For stomatal traits: we used imprinting method to measure stomatal traits (Mediavilla et al., 2021). First, we took the sample leaves out of the buffered FAA fixative solution and let them dry naturally. Next, we applied a transparent nail polish, roughly 1 mm² in area, to the lower epidermis of the leaves. Once the nail polish was fully dried, we used sharp tweezers to carefully peel it off and prepared temporary sections for further analysis. Observe and capture images under a microscope with a 20x eyepiece. For each slice, randomly select 3 fields of view and count the stomata in each, and then use Image J software to randomly choose 3 stomata to measure the SL and stomata width (SW) of their guard cells. The final SL and SW were calculated as the average of the guard cell’s length and width. SPI was calculated using the formula: SPI = SL² × SD × 10^-4^.

The leaf trait information was shown in **Supplementary Table S1**.

### 2.4 Environmental factors measurements

For canopy openness: For each sample tree, a fisheye camera (Nikon Coolpix 4500 digital camera with a 180°fisheye lens, Nikon Corp, Tokyo, Japan) was utilized to capture three hemispherical images for each sample tree, specifically in the south and north directions. Then, we utilized the Gap Light Analyzer ver.2.0 software to compute the canopy openness, which was used to determine the lighting conditions under which the sample was located (Frazer et al., 2000).

For each soil sample, we measure the soil water content using the drying method, which calculates the ratio of the soil’s dry weight to its wet weight. After drying the soil, we determine its pH using a HANNAPH2l1 pH meter. The soil total nitrogen content was determined using a Kjeldahl nitrogen analyzer (Jinan Hanon Instruments Co., Ltd., China). For the soil total phosphorus content, the molybdenum-antimony colorimetric method was employed. The information on environmental factors was shown in **Supplementary Table S1**.

### 2.5 Statistical analysis

All statistical analyses were conducted using R–4.2.1. Except for phyllotaxy 1, all other phyllotaxies were the average of the left and right leaves of the same phyllotaxy. The Least Significant Differences (LSD) method was applied to test for significant variations in single traits across different phyllotaxies. Principal component analysis (PCA) was conducted to extract the first principal component (PC) scores for economic and stomatal traits across different phyllotaxies. All trait variables were standardized using Z–score transformation prior to principal component analysis (PCA) to meet the assumption of normality. LSD was used to evaluate potential significant differences in the first PC scores of these traits between phyllotaxies. The standardized major axis (SMA) method was employed to examine the correlation between the two trait dimensions (Li et al., 2015). If the correlation slope between these two dimensions remained stable across phyllotaxies, the common slope was computed and compared to 1.00 to assess whether the traits followed an allometric growth relationship.

Then, a General Linear Model (GLM) was used to evaluate the effects of phyllotaxy, light intensity, and soil factors (including soil nitrogen, phosphorus, and water content) on the principal component scores of economic and stomatal traits. To prevent collinearity from distorting the model’s accuracy, the variance inflation factor (VIF) was calculated for each variable pair. If all VIF values were below 10, this would indicate that collinearity is not a concern (Dormann et al., 2013). The figures and tables were completed in R–4.2.1, Sigmaplot 10.0, and Excel 2021, respectively.

## 3 Results

### 3.1 Economic and stomatal traits vary with phyllotaxy

**Figure 2:**
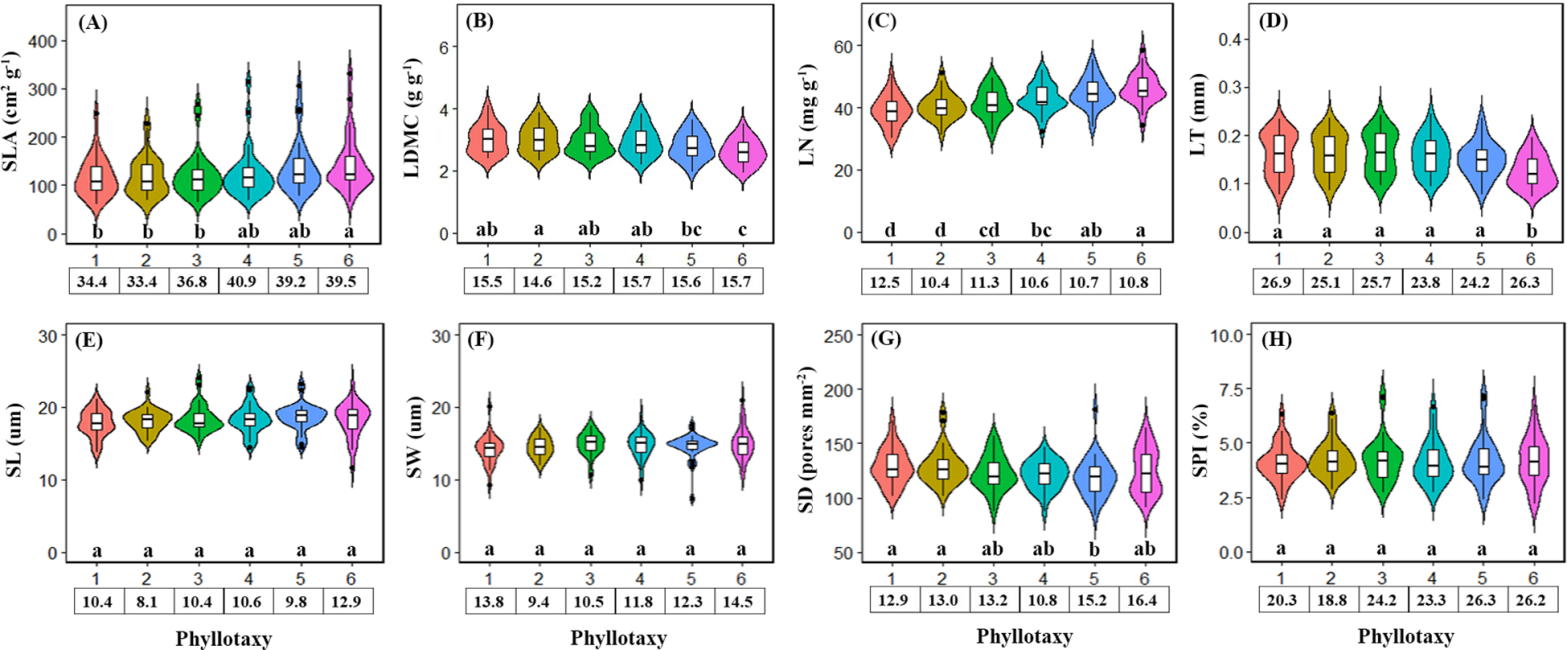
Leaf economics traits **(A-D)** and stomatal traits **(E-H)** varied with phyllotaxy. Different lowercase letters indicate a significant difference among phyllotaxy (*p* < 0.05). The numbers in the box below the figure are the coefficient of variation of the traits corresponding to the phyllotaxy.

The variation in economic traits with phyllotaxy was stronger than that of stomatal traits (**Figure 2**). For economic traits, SLA and LN increased significantly with phyllotaxy (**Figures 2A, C**), while LDMC and LT decreased (**Figures 2B, D**). For stomatal traits, SL, SW, and SPI showed no significant variation across phyllotaxies (**Figures 2E, F, H**), but SD decreased significantly (**Figure 2G**).

### 3.2 The correlation between leaf economics and stomatal traits varies significantly with phyllotaxy

**Figure 3:**
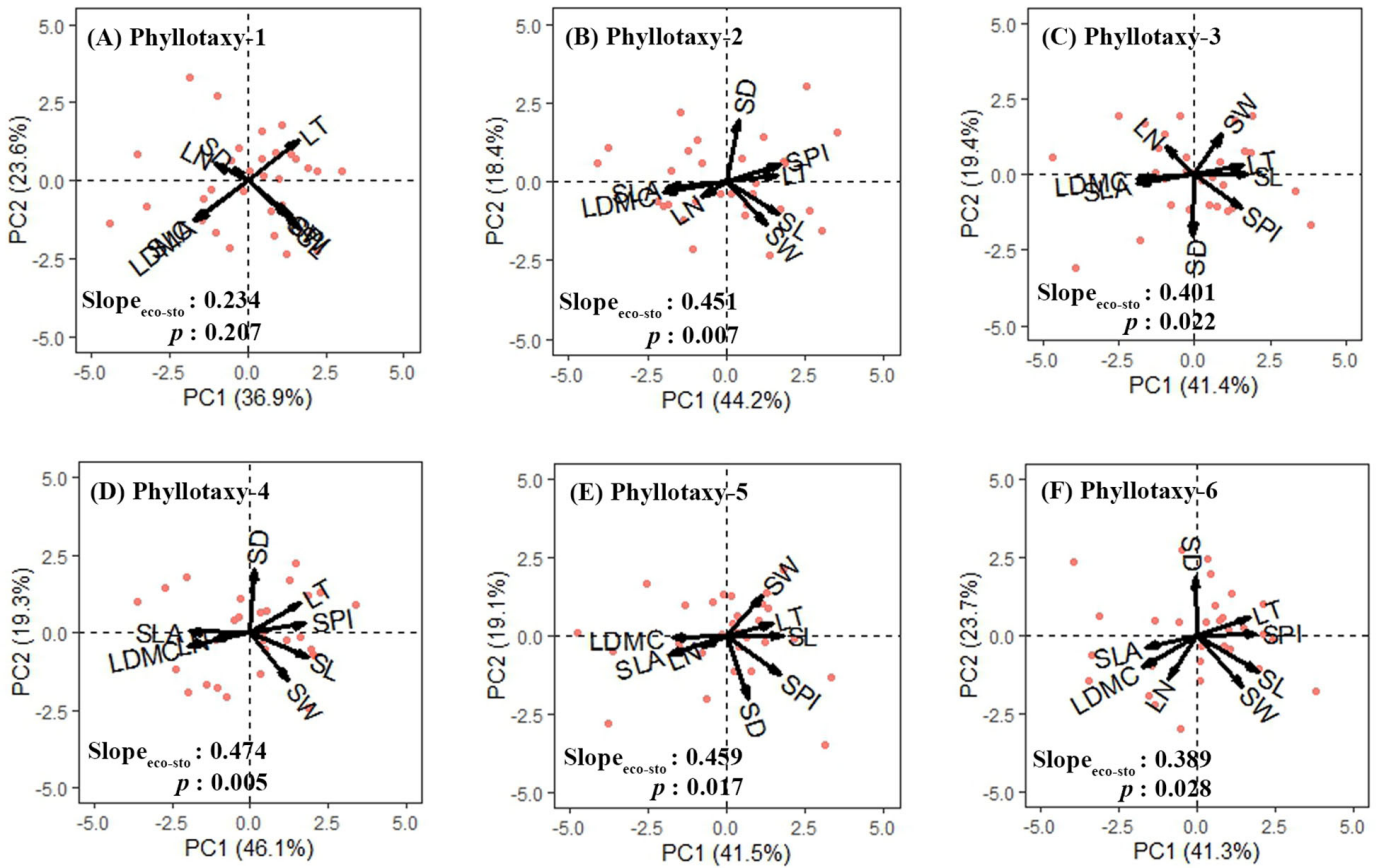
Principal component analysis of eight leaf traits for phyllotaxy 1-6 **(A–F)**. Arrows represent the principal component loadings associated with the leaf traits. The proportion of the total variation explained by the first two components is shown in parentheses next to the axis label. The first principal component scores of economic traits and stomatal traits were used to analyze the correlation between economic and stomatal traits of each phyllotaxy. Leaf trait abbreviations are provided in **Table 1**.

**Figure 4:**
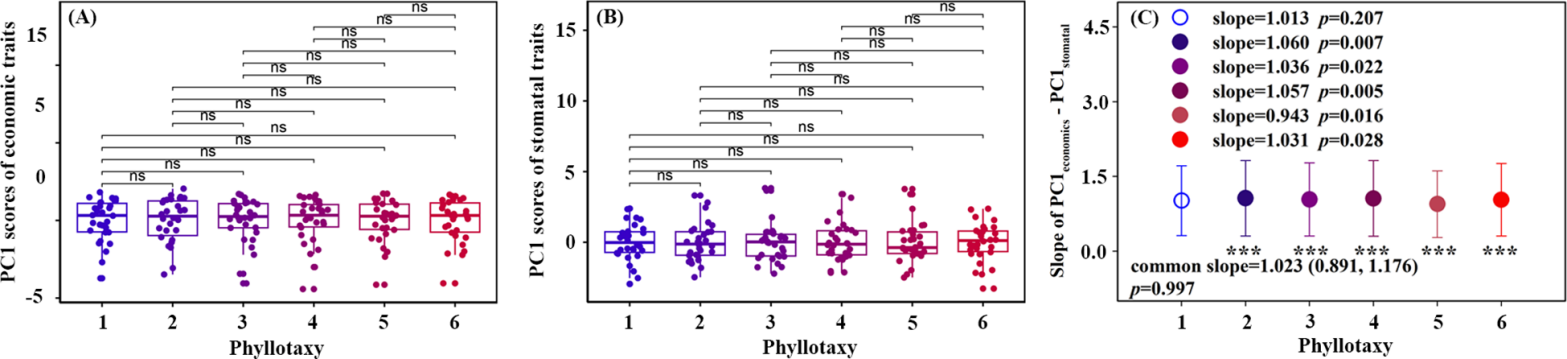
Phyllotaxy differences **(A, B)** and correlation **(C)** in the first principal component scores of economics traits and stomatal traits. Hollow circle indicates that there was no significant correlation between the first principal component scores of economic traits and stomatal traits (*p* > 0.05). The asterisk represents a significant difference between the slope and 1 (*p* < 0.05).

For the six phyllotaxies, the first two axes of the PCA explained 60.5%-65.4% of the variation in leaf traits (**Figure 3**). Axis 1 primarily represented economic traits variation, with strong loadings on SLA and LDMC, while axis 2 primarily represented stomatal traits variation, with significant loadings on SD and SW (**Table 1**). A decoupled relationship between economic and stomatal trait PC1 scores was observed at phyllotaxy 1 (*p* = 0.207), but a coupled relationship at phyllotaxies 2-6 (*p* < 0.05) (**Table 1**, **Figure 4C**, **Supplementary Figure S1**). PC1 scores for both economic and stomatal traits showed no significant differences across any pair of phyllotaxies (**Figures 4A, B**).

**Table 1:** Principal component analysis (PCA) results for the eight leaf traits for different phyllotaxy.

	Phyllotaxy-1		Phyllotaxy-2		Phyllotaxy-3		Phyllotaxy-4		Phyllotaxy-5		Phyllotaxy-6	
	PC1	PC2	PC1	PC2	PC1	PC2	PC1	PC2	PC1	PC2	PC1	PC2
Eigenvalue	2.94	1.89	3.53	1.46	3.31	1.55	3.69	1.54	3.32	1.53	3.3	1.9
Variation explained (%)	36.90%	23.60%	44.20%	18.40%	41.40%	19.40%	46.10%	19.30%	41.50%	19.10%	41.30%	23.70%
LT	0.41	0.40	0.38	0.07	0.39	0.10	0.36	0.33	0.36	0.15	0.38	0.18
LDMC	-0.41	-0.39	**-0.47**	-0.13	**-0.45**	-0.05	**-0.44**	-0.15	**-0.42**	-0.01	-0.40	-0.30
SLA	**-0.44**	-0.39	**-0.44**	-0.07	**-0.46**	-0.11	**-0.44**	0.01	**-0.45**	-0.21	-0.38	-0.11
LN	-0.25	0.17	-0.17	-0.18	-0.23	0.32	-0.27	-0.08	-0.19	-0.12	-0.21	**-0.42**
SW	0.33	-0.35	0.30	**-0.50**	0.22	**0.46**	0.28	**-0.53**	0.27	**0.46**	0.33	**-0.49**
SL	0.41	**-0.47**	0.39	-0.38	**0.43**	0.00	0.41	-0.28	**0.43**	0.00	**0.45**	-0.35
SD	-0.11	0.13	0.09	**0.72**	-0.01	**-0.71**	0.03	**0.70**	0.16	**-0.72**	-0.01	**0.57**
SPI	0.35	-0.37	0.41	0.20	0.38	-0.40	0.40	0.10	**0.42**	**-0.44**	**0.43**	0.02

For each axis, the eigenvalues, variance explained, and trait loadings on the first two components are presented. Eigenvectors with values greater than 0.41 are highlighted in bold. LT, leaf thickness; LDMC, leaf dry matter content; SLA, specific leaf area; LN, leaf nitrogen concentration; SW, stomatal width; SL, stomatal length; SD, stomatal density; SPI, stomatal pore indexpotential conductance index.

### 3.3 Phyllotaxy and environmental factors exert distinct influences on the variation of economic and stomatal traits

Phyllotaxy and environmental factors exhibited divergent effects on the PC scores of economic and stomatal traits (**Table 2**). Economic traits were significantly influenced by canopy openness, soil nitrogen, and phosphorus content (*p* < 0.05), whereas phyllotaxy and soil water content showed no significant effects (*p* > 0.05). In contrast, stomatal traits were primarily shaped by phyllotaxy (*p* < 0.05), while environmental variables, including canopy openness and soil nutrients, had no significant influence (*p* > 0.05).

**Table 2:** The influence of phyllotaxy and environmental factors on PC scores of economics and stomatal traits by using a GLM.

	PC scores of economics traits		PC scores of stomatal traits	
	Estimate Std.	*s* value	Estimate Std.	*s* value
Canopy openness	0.062	<0.001***	-0.003	0.741
Phyllotaxy	0.019	0.771	-0.193	<0.001***
Soil total nitrogen	0.189	0.038*	-0.109	0.115
Soil total phosphorus	-2.022	<0.001***	-0.049	0.904
Soil water content	0.748	0.447	0.599	0.426
Intercept	-1.399	0.021*	1.339	0.004**

## 4 Discussion

### 4.1 Single leaf economic traits showed stronger variation with phyllotaxy than stomatal traits

Our results clearly demonstrate that single leaf economic traits are more sensitive to changes in phyllotaxy within a compound leaf than single stomatal traits, highlighting the key role of phyllotaxy in regulating leaf resource acquisition and water use efficiency (**Figure 2**). For single economic traits, from the top to the bottom of the compound leaf (phyllotaxy 1–6), SLA and LN progressively increase, while LDMC and LT decrease (**Figures 2A-D**). SLA and LN are reliable indicators of leaf photosynthetic capacity, with higher values commonly observed in low-light environments and shade leaves (Liu et al., 2019; Bai et al., 2024). For compound leaves, the terminal leaflets (phyllotaxy–1 direction) are more exposed to sunlight, whereas the bottom leaflets (phyllotaxy-6 direction) are more shaded, leading to a reduction in ambient light density (Koch et al., 2018; Guo et al., 2025), typical of shade-adapted leaf traits. The higher SLA of the bottom leaflets may be related to a reduction in palisade tissue and an increase in spongy tissue, which enhances light transmittance and improves light energy capture efficiency (Poorter et al., 2019; Garnier et al., 2025). Additionally, thinner cell walls and larger intercellular spaces in the leaves allow for higher water retention, contributing to the observed decrease in LDMC and LT (John et al., 2013; Dastpak et al., 2025).

For single stomatal traits, our results did not show the high level of variation observed in large-scale studies (Xie et al., 2022; Liu et al., 2023), SL, SW, and SPI did not vary significantly across phyllotactic positions, but SD decreased significantly with phyllotaxy (**Figures 2E–H**). This could be due to the fact that lower SD reduces the leaf’s obstruction to light, enabling more light to penetrate the leaf and reach the mesophyll tissues (Terashima et al., 2011; Liu et al., 2018), thereby enhancing the photosynthetic efficiency of the lower leaflets of compound leaves. Additionally, Leaflets at lower phyllotactic positions (e.g., phyllotaxy 5–6) are often located in shaded zones of the canopy, where light intensity and vapor pressure deficit (VPD) are reduced. This microenvironmental condition leads to lower transpirational demand, which may explain the observed decline in stomatal density (SD) in basal leaflets.

### 4.2 Economic trait and stomatal trait dimensions exhibit allometric growth relationships within compound leaves

Our results clearly demonstrate that, except for phyllotaxy 1, the leaflets in phyllotaxies 2–6 exhibit independent dimensions for both economic traits and stomatal traits, implying that strategies for leaf resource acquisition and water use may be independently regulated within local microenvironments (**Figure 3**, **Table 1**). Furthermore, we also observed that the clustering of economic and stomatal traits became less pronounced in leaflets from phyllotaxies 5 and 6, suggesting that under conditions of strong environmental stress, plants may maintain a steady state of resource-water use through functional integration (such as synchronous adjustment of SLA and SPI) rather than relying on flexible adjustments of independent traits, which is in agreement with those of García-Cervigón et al. (2021).

It is noteworthy that, despite most traits show significant univariate variations observed among different phyllotaxies (**Figure 2**), no significant differences were found in the comprehensive trait dimensions extracted through principal component analysis across these phyllotaxies (**Figures 4A, B**). Our results are inconsistent with those of Boisseaux et al. (2024), whose study indicated that trait dimensions exhibit a stronger response to environmental factors than single traits. This discrepancy is likely due to their study being conducted at the interspecific level, while ours focused on intraspecific variation. Our results suggest that compound-leaved species may exhibit high plasticity in specific functions but maintain relative stability in their overall functional strategies. This phenomenon may be attributed to the dimensionality reduction analysis method, which compresses the covariation information of multiple traits into a few principal components (i.e., integrating multiple independent traits into functional dimensions such as “resource acquisition dimension” and “water use dimension”), with trait variations offsetting each other in the principal component dimensions. In addition, single traits respond directly to environmental signals (such as changes in SLA induced by light intensity), thus exhibiting high levels of variation; in contrast, the integrated dimensions reflect the synergistic relationships among traits, which may lead to some of the variations in independent traits being “masked.” This could also be a reason for the non-significance of trait dimensions among phyllotaxies. The research findings reveal a multi-level regulatory mechanism in plants for plasticity in overall functional strategies and local traits when adapting to complex environments.

The correlation between economic and stomatal trait dimensions varies significantly with phyllotaxy: In phyllotaxy 1, the two sets of traits demonstrated a decoupled yet isometric relationship in their growth (*p* > 0.05, **Figure 4C**), suggesting that the variations in these two functional trait dimensions occurred independently of each other. This pattern likely suggests that under stable, low-stress environmental conditions, plants can independently regulate resource acquisition and water use functions (Li et al., 2015; Liu et al., 2020). In phyllotaxies 2–6, a significant coupling and allometric growth relationship was observed between economic and stomatal trait dimensions (*p* < 0.05, **Figure 4C**), indicating a coordinated regulation of resource acquisition and water use functions in leaves (Xu et al., 2021). The decoupling relationship at phyllotaxy 1 may result from developmental priority in terminal leaflets, while coupling in phyllotaxies 2–6 suggests integrated regulation under shared microenvironments. Further analysis revealed a common slope between the two trait groups (*p* = 0.997, common slope = 1.023, **Figure 4C**), suggesting that phyllotaxy does not significantly affect their relationship. Additionally, the coordination between the traits exhibited a slope greater than 1, indicating that as stomatal traits intensify, economic traits increase even more. This reflects a tendency in plants from lower and middle phyllotaxies to enhance carbon acquisition to offset physiological declines under limited light or transpiration, embodying a resource-acquisition–focused regulatory strategy (Tang et al., 2022).

### 4.3 Phyllotaxy and environmental factors have differential effects on leaf economic and stomatal trait dimensions

Understanding the interactions between phyllotaxy, environmental factors, and plant traits is crucial for revealing how plants optimize their functional strategies across varied ecological contexts (Boisseaux et al., 2024; Guo et al., 2025). Although the LSD test results did not reveal any significant patterns of variation in economic and stomatal trait dimensions across phyllotactic levels (**Figures 4A, B**), the GLM analysis, after controlling for environmental variables, demonstrated that phyllotaxy and environmental factors exerted significant and distinct effects on these two sets of trait dimensions (**Table 2**). These responses are likely driven by the functional divergence between the two trait categories, reflecting the multifunctionality and trade-off strategies plants use to adapt to complex environmental conditions. Economic traits are tightly associated with plant strategies for resource acquisition, allocation, and growth, making them highly responsive to environmental factors such as light availability and soil nutrients (Joswig et al., 2022; Liu et al., 2024). Our findings show that canopy openness and soil nitrogen positively influence economic trait dimensions. This suggests that, under high light and nitrogen availability, plants tend to adopt a fast-growth strategy to boost photosynthetic efficiency and biomass production (Jager et al., 2015; Singh and Verma, 2025). This pattern is consistent with the resource acquisition strategy framework proposed by the leaf economics spectrum (Wright et al., 2004). However, the significant negative impact of soil phosphorus content on economic trait dimensions (*p* < 0.001) likely indicates that plants are employing a more conservative resource allocation strategy under current environmental conditions, such as decreasing growth rate to enhance resilience, in their uptake of soil phosphorus (Han et al., 2021). No significant association was found between soil water content and economic trait dimensions (*p* = 0.447), possibly due to the indirect role of water availability in regulating plant growth and resource allocation, or because plants may compensate for water variability through alternative mechanisms, such as stomatal regulation.

Our results indicate that stomatal trait dimensions are primarily influenced by phyllotaxy, with weaker responses to light and soil nutrients (**Table 2**). Stomatal traits play a key role in regulating plant transpiration and gas exchange, with their aperture dynamics largely governed by the plant’s internal water status and physiological signals such as abscisic acid (Liu et al., 2021). For compound leaves, phyllotaxy can optimize the distribution pattern of stomata on the leaf surface, regulating stomatal behavior to effectively reduce unnecessary water loss, ensuring adequate and efficient gas exchange (Yang et al., 2019). While light and soil nutrients are critical for plant growth and photosynthesis, their effect on stomatal traits is more indirect, primarily influencing the plant’s photosynthetic efficiency and nutrient absorption capacity, thereby indirectly affecting stomatal regulation rather than directly altering stomatal opening and closing patterns. In summary, the strong correlation between economic traits and environmental factors-such as light availability and soil nitrogen-highlights the plasticity of plant resource acquisition strategies, while the dependence of stomatal traits on phyllotaxy illustrates the conservative strategy of plants in maintaining water balance through structural-physiological coupling. Moreover, although this study reveals patterns of trait variation and coordination along the phyllotactic developmental gradient and evaluates the respective influences of phyllotaxy and environmental factors on trait expression, further investigation is required to determine the extent to which these patterns are consistent across diverse species and ecological contexts.

## 5 Conclusion

Our findings clearly show that phyllotaxy significantly regulates single traits but does not influence comprehensive trait dimensions, highlighting the high plasticity of compound leaf plants in specific functions and the stability of their overall functional strategies. Economic and stomatal trait dimensions are decoupled in phyllotaxy 1, but coupled in phyllotaxies 2–6, indicating that the integration of these traits may vary across leaf positions in response to both environmental and developmental factors. Furthermore, phyllotaxy significantly influences the variation in stomatal trait dimensions, while environmental factors primarily drive the variation in economic trait dimensions. We suggest that the role of phyllotaxy in shaping trait and their relationships should be given more attention in future trait-based ecological studies. Incorporating phyllotaxy into future research will offer deeper insights into the mechanisms driving plant functional differentiation and ecological adaptability across varying environments.

## Data Availability

The raw data supporting the conclusions of this article will be made available by the authors, without undue reservation.
