# Supplementary material for: Phyllotaxy and environmental factors influences on leaf trait dimensions in Fraxinus mandshurica: a multidimensional approach within temperate forests
Source: Front Plant Sci. 2025 Jul 15;16:1626579. doi: 10.3389/fpls.2025.1626579 (PMC12303895; doi:10.3389/fpls.2025.1626579)

**Supplementary files**

**TABLE S1** Information of 8 leaf traits and 4 environmental factors.

|  | Abbreviation | Unit | Mean | Max | Min | SE |
| --- | --- | --- | --- | --- | --- | --- |
| **Leaf traits** |  |  |  |  |  |  |
| Specific leaf area | SLA | cm^2^ g^-1^ | 127.06 | 331.01 | 61.16 | 3.68 |
| Leaf dry matter content | LDMC | g g^-1^ | 2.89 | 4.09 | 1.95 | 0.04 |
| Leaf thickness | LT | mm | 0.15 | 0.24 | 0.07 | 0.003 |
| Leaf nitrogen concentration | LN | mg g^-1^ | 42.53 | 58.53 | 30.27 | 0.39 |
| Stomatal length | SL | um | 18.27 | 24.11 | 11.64 | 0.14 |
| Stomatal width | SW | um | 14.68 | 21.04 | 7.39 | 0.13 |
| Stomatal density | SD | pores mm^-2^ | 124.34 | 181.86 | 83.94 | 1.31 |
| Stomatal pore index | SPI | % | 4.18 | 7.16 | 2.18 | 0.07 |
| **Environmental factors** |  |  |  |  |  |  |
| Soil water content | SWC | % | 0.56 | 0.79 | 0.33 | 0.01 |
| Soil total nitrogen | STN | mg g^-1^ | 7.87 | 14.21 | 3.86 | 0.16 |
| Soil total phosphorus | STP | mg g^-1^ | 1.12 | 2.11 | 0.56 | 0.03 |
| Canopy openness | CO | % | 27.42 | 47.22 | 13.56 | 0.77 |

**Figure legends**

**Figure S1** Principal component analysis of four economic traits (A-F) and four stomatal traits (G-L) for different phyllotaxy. Arrows represent the principal component loadings associated with the leaf traits. The proportion of the total variation explained by the first two components is shown in parentheses next to the axis label. Leaf trait abbreviations are provided in Table 1.

**Figure S1**


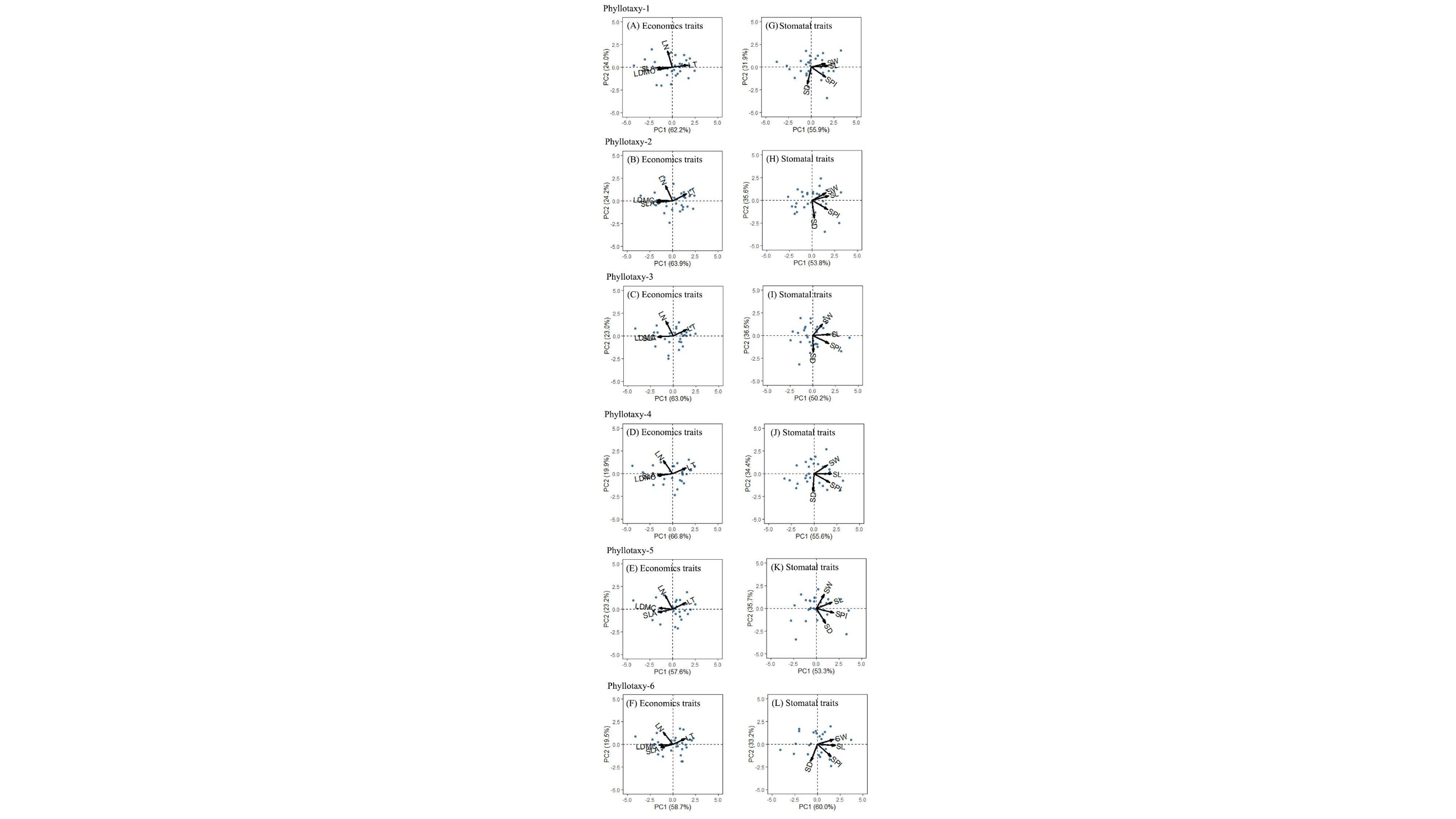

Supplement: Supplementary file 1 [file DataSheet1.docx]
